# Identification of Protein Biomarkers for Differentiating *Listeria monocytogenes* Genetic Lineage III

**DOI:** 10.3390/foods13091302

**Published:** 2024-04-24

**Authors:** Basant Gomaa, Jingjun Lu, Hossam Abdelhamed, Michelle Banes, Olga Pechanova, Tibor Pechan, Mark A. Arick, Attila Karsi, Mark L. Lawrence

**Affiliations:** 1 Department of Comparative Biomedical Sciences, College of Veterinary Medicine, Mississippi State University, Mississippi State, MS 39762, USA; bmg381@msstate.edu (B.G.); jingjun.lu@stjude.org (J.L.); abdelhamed@cvm.msstate.edu (H.A.); banes@cvm.msstate.edu (M.B.); karsi@cvm.msstate.edu (A.K.); 2 Institute for Genomics, Biocomputing, and Biotechnology, Mississippi State University, Mississippi State, MS 39762, USA; olga@igbb.msstate.edu (O.P.); pechan@ra.msstate.edu (T.P.); maa146@igbb.msstate.edu (M.A.A.II)

**Keywords:** *Listeria monocytogenes* NRRL B-33077, genetic lineage III, mass spectrometry, protein biomarker

## Abstract

*Listeria monocytogenes* is the causative agent of listeriosis, a severe foodborne illness characterized by septicemia, meningitis, encephalitis, abortions, and occasional death in infants and immunocompromised individuals. *L. monocytogenes* is composed of four genetic lineages (I, II, III, and IV) and fourteen serotypes. The aim of the current study was to identify proteins that can serve as biomarkers for detection of genetic lineage III strains based on simple antibody-based methods. Liquid chromatography (LC) with electrospray ionization tandem mass spectrometry (ESI MS/MS) followed by bioinformatics and computational analysis were performed on three *L. monocytogenes* strains (NRRL B-33007, NRRL B-33014, and NRRL B-33077), which were used as reference strains for lineages I, II, and III, respectively. Results from ESI MS/MS revealed 42 unique proteins present in NRRL B-33077 and absent in NRRL B-33007 and NRRL B-33014 strains. BLAST analysis of the 42 proteins against a broader panel of >80 sequenced strains from lineages I and II revealed four proteins [TM2 domain-containing protein (NRRL B-33077_2770), DUF3916 domain-containing protein (NRRL B-33077_1897), DNA adenine methylase (NRRL B-33077_1926), and protein RhsA (NRRL B-33077_1129)] that have no homology with any sequenced strains in lineages I and II. The four genes that encode these proteins were expressed in *Escherichia coli* strain DE3 and purified. Polyclonal antibodies were prepared against purified recombinant proteins. ELISA using the polyclonal antibodies against 12 *L. monocytogenes* lineage I, II, and III isolates indicated that TM2 protein and DNA adenine methylase (Dam) detected all lineage III strains with no reaction to lineage I and II strains. In conclusion, two proteins including TM2 protein and Dam are potentially useful biomarkers for detection and differentiation of *L. monocytogenes* lineage III strains in clinical, environmental, and food processing facilities. Furthermore, these results validate the approach of using a combination of proteomics and bioinformatics to identify useful protein biomarkers.

## 1. Introduction

*Listeria monocytogenes* is a Gram-positive intracellular pathogen that causes listeriosis, a serious foodborne infection with high hospitalization and mortality rates (20 to 30% mortality in high-risk individuals) [1,2,3]. In the United States, *L. monocytogenes* is responsible for about 260 deaths each year. Foodborne outbreaks of listeriosis are serious public health problems and can cause severe clinical illness in high-risk populations, with listeriosis having the third highest mortality rate among foodborne pathogens. The most common form of the disease occurs 24 h after ingestion of a large inoculum of bacteria and usually lasts two days with gastroenteritis-like symptoms [4]. The less common but more severe form of the disease is found mainly among pregnant women and immunocompromised persons, with symptoms of abortion, neonatal death, septicemia, and meningitis [5].

*L. monocytogenes* is ubiquitous in the environment, including water and soil, and it can resist high salt concentration, low and high temperatures (−1 to 45 °C), and a broad pH range (4.6 to 9.5) [6,7]. This capacity for tolerating extreme conditions makes *L. monocytogenes* of particular concern for the food industry, especially because it can be carried asymptomatically by multiple animal species [8]. As a result, it has been isolated from raw and processed foods, including chicken, red meat, seafood, and dairy products.

Based on classical molecular subtyping methods such as ribotyping, pulse field gel electrophoresis (PFGE), and multilocus sequence typing (MLST), *L. monocytogenes* strains are grouped into four distinct phylogenetic lineages (I, II, III, and IV). Lineage I includes strains of serotypes 1/2b, 3b, 4b, 4d, and 4e; this lineage is more commonly associated with human clinical cases. Most outbreaks of listeriosis are caused by this lineage. Lineage II includes strains of serotypes 1/2a, 1/2c, 3a, and 3c [9], which is mostly isolated from food and environmental samples and causes sporadic listeriosis. Lineage III includes strains of serotypes 4a and 4c, as well as certain strains of serotype 4b commonly isolated from animals. Strains in this lineage are typically lower risk for causing listeriosis in people [10,11,12]. Lineage IV strains are very rare and mostly come from animals [13].

*L. monocytogenes* can be differentiated through culture methods based on selective enrichment and plating, followed by characterization based on colony morphology, sugar fermentation, and hemolytic properties [14]. These methods are the gold standard, but they are lengthy and may not be suitable for testing foods with short shelf lives.

As a result, more rapid tests were developed based on molecular techniques (PCR or DNA hybridization). However, some PCR techniques require two or three independent PCR reactions [15,16]. Other PCR techniques differentiate only two or three serotypes [17,18]. Thus, there is a need for the development of a rapid method that can distinguish high-risk *L. monocytogenes* serotypes from low-risk serotypes. Since lineage III strains are low risk for human listeriosis, we sought to identify protein biomarker(s) that could distinguish *L. monocytogenes* genetic lineage III isolates from lineage I and II isolates using a simple antibody-based method. This work will enable studies on the epidemiology of listeriosis and the development of preventive strategies.

In the current study, we applied liquid chromatography and electrospray ionization with tandem mass spectrometry (ESI MS/MS) proteomics to identify unique proteins of *L. monocytogenes* lineage III strain NRRL B-33077. Mass spectrometry identified a total of 42 distinct proteins in strain NRRL B-33077 with no ortholog in representative strains from *L. monocytogenes* genetic lineages I and II. BLAST analysis of these lineage III-unique proteins against a panel of 80 *L. monocytogenes* proteomes (representing lineages I, II, and III) revealed twenty proteins without protein homology with lineage I and II proteins. Four proteins out of twenty were chosen for further studies based on peptide coverage and identity. These proteins have potential to develop antibody-based separation methods for differentiating *L. monocytogenes* genetic lineage III from lineages I and II. Development of simple antibody-based methods for differentiating *L. monocytogenes* genetic lineages, serotypes, and epidemic clones would facilitate identification of listerial subgroups in diagnostic microbiology laboratories and potentially in food processing facilities. This increased discriminatory capability would assist in assessment of risk from *L. monocytogenes* isolates and accelerate epidemiological investigations.

## 2. Materials and Methods

### 2.1. Ethics Statement

Polyclonal antibody production in rabbits was performed at Mississippi State University according to a protocol approved by the MSU Institutional Animal Care and Use Committee (IACUC-18-137).

### 2.2. Bacterial Cultures

Twelve *L. monocytogenes* strains were used in this study as reference strains for lineages I, II, and III (Table 1). *L. monocytogenes* genome sequence for strains NRRL B-33007, NRRL B-33014, and NRRL B-33077 was provided by Todd J. Ward. *L. monocytogenes* strain 33077 (genomovar III; serotype 4a) was obtained from USDA-ARS and used for development of a DNA probe-based method for differentiation of *L. monocytogenes* serogroups [12]. Strain NRRL B-33077 served as the reference genetic lineage III strain for the current study. *L. monocytogenes* strains were grown on brain heart infusion (BHI) agar and broth (Difco, Sparks, MD, USA) and incubated at 37 °C. *Escherichia coli* K12 strain NovaBlue (Novagen, Madison, WI, USA) was used for gene cloning in pET-28a expression vector (Novagen) (Table 2). Three recombinant proteins (WP_012582068.1, WP_003728958.1, and WP_014589151.1) were expressed in BL21(DE3) (EMD Millipore, San Diego, CA, USA), and one protein (WP_077954308.1) was expressed in C41(DE3) (EMD Millipore). All *E. coli* strains were cultured on Luria–Bertani (LB) agar or broth (Difco) at 37 °C. Kanamycin (Kan: 30 µg/mL) (Sigma–Aldrich, St. Louis, MO, USA) was added to a culture medium for plasmid selection when needed, and isopropyl-β-D thiogalactopyranoside (IPTG, Sigma) was added for protein induction when needed.

### 2.3. Protein Extraction with Phenol-Based Protocol

Total proteins were isolated in triplicate from *L. monocytogenes* strains NRRL B-33007, NRRL B-33014, and NRRL B-33077 (lineages I, II, and III, respectively) using a phenol-based extraction protocol with modifications [21]. Washed *L. monocytogenes* pellets were resuspended in an ice-cold extraction buffer (0.9 M sucrose, 0.5 M Tris-base, 0.05 M Na2-EDTA, 0.1 M KCl, 2% β-mercaptoethanol, 8 mM PMSF, pH 8.7) and sonicated with five 10-s pulses (Fisher Scientific Model 100 Sonic Dismembrator, setting 3) on ice with a minimum of 1 min cooling time between pulses. Lysates were further treated with DNase (85 µg/mL) and RNase (20 µg/mL) at 37 °C for 30 min. Proteins were extracted by adding an equal volume of Tris-buffered phenol (pH 8) and homogenizing for 10 min at room temperature. Insoluble matter, phenol, and aqueous phases were separated by centrifugation at 5500× *g* at 4 °C. The phenol phase was collected and extracted with an ice-cold extraction buffer two more times using the same procedure. Proteins were then precipitated from the phenol phase with five volumes of 0.1 M ammonium acetate and 2% β-mercaptoethanol in 100% methanol overnight at −20 °C.

Precipitated proteins were washed three times with the same solution and three times with 100% acetone. Air-dried protein pellets were stored at −20 °C.

### 2.4. Protein Fractionation and In-Solution Digestion

For protein fractionation and digestion, *L. monocytogenes* protein pellets were dissolved in 1× SDS sample buffer (2% SDS, 50 mM Tris-HCl pH 6.8, 10% glycerol; 1% DTT) and quantified using a 2D Quant Kit (GE Healthcare, Chicago, IL, USA). To increase coverage of each proteome, 350 µg of proteins were fractionated into twelve molecular weight (MW) fractions using Sage ELF (Sage Science, Beverly, MA, USA). After fractionation, protein purity was verified using Bioanalyzer (Agilent Technologies, Santa Clara, CA, USA). Fractions were desalted on HiPPR spin columns (Thermo Fisher Scientific, Waltham, MA, USA). In-solution digestion was conducted as follows: each fraction was reduced with 100 mM DTT (15 min at 65 °C), alkylated with 100 mM iodoacetamide (IAA) 30 min at room temperature in the dark, and digested overnight with Trypsin/Lys-C Mix (Promega, Madison, WI, USA) at 37 °C. Tryptic peptides were acidified with formic acid, concentrated by Speed-Vac, and stored at −80 °C. Immediately before mass spectrometry, they were dissolved in 2% acetonitrile/0.1% formic acid and subjected to LC-MS/MS. Protein fractionation was performed at Institute for Genomics, Biocomputing and Biotechnology (IGBB-MSU).

### 2.5. Mass Spectrometry

Trypsin-digested protein fractions from three *L. monocytogenes* strains were subjected to qualitative proteomics analysis using liquid chromatography and ESI MS/MS. Peptides were separated using C18 column and HPLC directly linked to an LTQ Orbitrap mass spectrometer. Mass spectra were collected in data-dependent analysis (DDA) mode during a three hour-long acetonitrile gradient. Complete open reading frames (ORFs) and translated proteins were predicted using EMBOSS (v 6.6.0).

The raw data files were converted to “MGF” format and matched against the ORF database using X!Tandem (v 2017.2.1.4). For each strain, search results from the strain-specific ORF database were filtered to reduce the false discovery rate to less than 5% using the MSnID R package. Results were filtered further by removing all spectra that matched theoretical ORF databases from NRRL B-33007 and NRRL B-33014 (genomovars I and II) to yield candidate genomovar III-specific spectra.

### 2.6. Bioinformatics Analysis

Bioinformatics analysis identified 232,470 spectra that were identified in NRRL B-33077 (lineage III) and not in lineages I and II. The unique spectra mapped to 1522 ORFs with an ORF FDR ≤ 0.05. Of those ORFs, a total of 42 distinct proteins were detected in strain NRRL B_33077 that did not match *L. monocytogenes* strains NRRL B-33007 and NRRL B-33014 with less than 50% identity.

BLAST analysis of the putative lineage III-specific proteins was conducted against 80 *L. monocytogenes* strains from lineages I and II in the NCBI Reference Sequence proteome database, and 20 proteins were identified that did not match any strains from lineages I and II.

Four of these 20 proteins, that were most unique to lineage III (based on peptide coverage and identity of all three lineages), are WP_012582068.1 encoding DUF3916 domain-containing protein (NRRL B-33077_1897, designated DUF3916), WP_003728958.1 encoding TM2 domain-containing protein (NRRL B-33077_2770, designated TM2), WP_014589151.1 encoding protein RhsA (NRRL B-33077_1129, designated RhsA), and WP_077954308.1 encoding Dam (NRRL B-33077_1926, designated Dam). These proteins were selected for further analysis to determine their suitability as biomarkers for antibody-based detection of *L. monocytogenes* genetic lineage III.

### 2.7. Construction of Recombinant Plasmids and Protein Expression

PCR amplicons for the selected four genes were amplified from *L. monocytogenes* NRRL B-33077 genomic DNA using the primer pairs shown in Table 3. Restriction endonuclease sites were incorporated at the 5′ end of each primer. The four amplified products were purified with the QIAquick PCR purification kit (Qiagen, Valencia, CA, USA). Cleaned PCR products were digested with two restriction endonucleases and ligated into pET28a. Ligation mixtures were transformed to chemically competent NovaBlue *E. coli* K12. Positive clones were selected on LB agar plates with kanamycin. Candidate plasmids with appropriate fragment patterns were sequenced using T7 promotor and T7 terminator primers to confirm correct orientation of the insert. Three resulting recombinant plasmids (pET28a_1897, pET28a_2770, and pET28a_1129) were transformed into *E. coli* BL21(DE3), while (pET28a_1926) was transformed into *E. coli* C41(DE3) due to the low level of expression in BL21 (DE3). Protein expression in *E. coli* cultures was optimized in 250 mL cultures and induced at an optical density at 600 nm (OD600) of 0.6–0.8 by adding IPTG ranging from 1 mM to 100 mM final concentration. Induced cultures were incubated overnight in temperatures ranging from 18 to 37 °C. Bacterial pellets at different time points (2, 4, 6, 8, and 18 h) were collected and analyzed by electrophoresis in 12% SDS-PAGE to check for protein expression. Non-recombinant bacteria and uninduced recombinant clones were used as controls [22].

### 2.8. Purification of Four Recombinant Proteins

Purification of the four recombinant proteins containing six histidine tags was carried out using His-Bind (Novagen) resin column according to the manufacturer’s protocols [22]. The recombinant DUF3916 and TM2 clones were grown in 250 mL of LB broth and induced by 1 mM final concentration IPTG for 18 h at 18 °C. Recombinant RhsA and Dam clones were grown in 250 mL of LB broth and induced by 100 mM IPTG. Bacteria were then harvested by centrifugation (6000× *g* for 20 min at 4 °C), and the pellets were lysed using 8 M urea, 50 mM Tris-HCl (pH 8.0), 10 mM EDTA, and 10 mg/mL lysozyme, followed by sonication (6 cycles, 30 s) on ice. The sonicated suspension of three proteins (DUF3916, TM2, and RhsA) was subjected to centrifugation and collected for protein purification. For Dam, the pellet was washed with 0.2 M sodium phosphate buffer (pH 7.3), 1 mM EDTA, 50 mM NaCl, 5% glycerol, and 1 M urea, followed by washing with a homogenization buffer (50 mM Tris-HCl (pH 8.0), 100 mM NaCl, 0.5% TritonX-100, 0.1% sodium-azide). The pellets were solubilized in 6 M guanidinium chloride, 10 mM Tris-HCl (pH 8.0), and 500 mM NaCl for 1 h at 4 °C followed by centrifugation. The clarified supernatant was loaded onto a His-Bind column prepacked with Ni^2+^-charged resin that had been pre-equilibrated with 10 mL of binding buffer. Non-specific proteins were removed by applying binding buffer followed by wash buffer (6 M urea, 500 mM NaCl, 20 mM imidazole, and 20 mM Tris-HCl [pH 7.9]). Recombinant proteins were then eluted with 6 M urea, 1 M imidazole, 250 mM NaCl, and 10 mM Tris-HCl. Purity of the proteins was determined by 12% SDS-PAGE analysis. Protein yield was determined on a spectrophotometer at 280 nm.

### 2.9. Polyclonal Antibody Production

A total of eight 10-week-old specific pathogen free (SPF) New Zealand white rabbits were housed separately in stainless steel cages and allowed free access to complete pelleted rabbit diet and water. Rabbits were acclimated for seven days before use. Two rabbits were used to produce polyclonal antibody against each recombinant protein.

On day 1, blood samples were collected from the ear vein of each rabbit to determine pre-injection antibody levels (control). Primary immunization with the purified proteins was conducted in 1 mL emulsion of sterile antigen and complete Freund’s adjuvant. The ratio of antigen to adjuvant was 4:1 with a final concentration of 250 µg/mL of recombinant protein. Rabbits were administered with 0.3 mL of the antigen/adjuvant mixture subcutaneously in four locations on the back and flank regions. Two booster immunizations were performed at 14- and 21-day intervals using emulsion with incomplete Freund’s adjuvant administered subcutaneously in four different locations. The ratio of antigen to adjuvant was 2:1. Post-injection blood samples were collected 7 and 21 days after the second booster injection to determine antibody titers using an enzyme-linked immunosorbent assay (ELISA).

After a sufficient antibody response was developed, a final blood sample was collected by the intracardiac (IC) method under anesthesia with ketamine (15 mg/kg), dexmedetomidine (0.125 mg/kg), butorphanol (0.2 mg/kg), and glycopyrrolate (0.01 mg/kg). Rabbits were euthanized without recovery from anesthesia using dexmedetomidine reversed with atipamezole (as needed).

### 2.10. Analysis of Antibody Titer

Analysis of antibody titer in rabbit serum was carried out using two different methods of the enzyme-linked immunosorbent assay (ELISA). In the first method, a 96-well ELISA plate (Bloomington, MN, USA) was coated with the purified recombinant proteins. The purified protein sample was diluted to 20 mg/mL final concentration using sterile PBS, and 100 μL was added per well. In the second method, a 96-well ELISA plate (Bloomington, MN, USA) was coated with inactivated bacterial suspension. *L. monocytogenes* strains (listed in Table 1) were cultivated to a concentration of 10^8^ CFU/mL, heat inactivated for 3 h, washed, and suspended in sterile Hank’s balanced salt solution (HBSS).

For both ELISA methods, 100 µL rabbit serum diluted 1:100 was added to each well. After washing, goat anti-rabbit IgG antibody conjugate (Fisher Scientific) was used for detection with p-nitrophenyl phosphate substrate (Sigma 104 phosphatase substrate) dissolved in 10% diethanolamine buffer. Absorbance was measured at 405 nm in an ELISA Microplate Reader (Carlsbad, CA, USA). To standardize, average background absorbance for each plate was subtracted from the measured absorbance for each well.

### 2.11. Automated Western Blot (WES)

Automated Western Blot (WES) immunoassays were conducted using a fully automated Simple WesternTM capillary instrument (Protein Simple, San Jose, CA, USA) according to the manufacturer’s protocol. For Dam, the assay was set up as follows: final concentration of 5 µg/mL per well of protein diluted in PBS. Antibodies against Dam were applied in dilutions of 1:250, 1:500, 1:750, and 1:1000 as one dilution per well. For TM2 protein, a final concentration of 2.5 µg/mL of protein diluted in PBS was used per well. Antibodies against Tm2 protein were applied in dilutions of 1:500, 1:1000, 1:2500, 1:5000, 1:7500, 1:10,000, and 1:15,000 as one dilution per well. Protein samples, antibodies, and reagents were loaded on pre-filled cartridges. Serum (primary antibody) and anti-rabbit HRP-conjugated secondary antibody were used for immunoprobing with chemiluminescent substrate, which took place in the capillaries. Resulting assay data were automatically processed and analyzed by Compass software (Protein Simple) (https://www.biotechne.com/resources/instrument-software-download-center, accessed on 22 March 2024).

### 2.12. Statistical Analysis

The rabbit serum antibody response against the injected recombinant proteins (measured by ELISA using plates coated with respective recombinant protein) was compared between the preinjected serum and the 21 day post-injection sera with mixed model logistic regression using PROC GLIMMIX in SAS for Windows 9.4 (SAS Institute, Inc., Cary, NC, USA).

The variance in the mean difference in reactivity between preinjected serum and immune serum for each protein against the mean of all four lineage I strains, four lineage II strains, and four lineage III strains was compared using PROC GLM Dunnett in SAS for Windows 9.4.

Additionally, a multiple variant comparison of the mean difference in reactivity for each genetic lineage III strain was carried out separately against the mean differences of all four lineage I and all four lineage II strains using PROC GLM with an adjustable *p* value.

## 3. Results

### 3.1. Mass Spectrometry

Liquid chromatography ESI MS/MS analysis revealed that 232,470 spectra were detected in *L. monocytogenes* NRRL B-33077 (lineage III) and absent in NRRL B-33007 and NRRL B-33014 (lineages I and II, respectively) with a false discovery rate (FDR) ≤ 0.05. These spectra mapped to 1522 ORFs, out of which 651 had unique peptides present in NRRL B-33077 and not in NRRL B-33014 and NRRL B-33007. Out of the 651 ORFs with unique peptides, 42 ORFs had no orthologs in NRRL B-33014 and NRRL B-33007.

These 42-candidate lineage III-specific proteins were identified based on a comparison of three strains (one from each genetic lineage). To determine which of these 42 have the best potential as biomarkers that would be uniquely present in all lineage III strains (and missing in all lineage I and II strains), these 42 proteins were analyzed by BLAST against the proteomes of 80 sequenced *L. monocytogenes* strains (representing all three genetic lineages) available in NCBI. Results revealed that 20 proteins out of 42 have less than 50% percent identity × percent coverage (%identity × %coverage) to any lineage I and II proteins (Table 4). The predicted function of the 20 candidate ORFs was determined and is shown in Table 5. Table 6 shows the number of peptides and the percent coverage of each ORF based on all unique peptides. Four proteins (TM2 domain-containing protein, DUF3916 domain-containing protein, Dam, and protein RhsA) out of twenty had the highest peptide coverage in lineage III strains and lowest identity and coverage in lineage I and II strains, and these four were selected for further analysis.

### 3.2. Expression and Purification of the Recombinant Proteins

Four genes encoding TM2 protein (NRRL B-33077_2770), DUF3916 domain-containing protein (NRRL B-33077_1897), Dam (NRRL B-33077_1926), and protein RhsA (NRRL B-33077_1129) from *L. monocytogenes* NRRL B-33077 were successfully cloned into pET28a vector, which was confirmed by restriction enzyme analysis and DNA sequencing. The result of expression analysis revealed that the induced recombinant bacteria started expression of TM2 and DUF3916 proteins after 6 h of induction with 1 mM final concentration of IPTG and reached maximum expression after 18 h at 18 °C. On the other hand, induced *E. coli* started expression of RhsA and Dam proteins after 2 h of induction with 100 mM IPTG and reached a maximum level at 6 h at 37 °C.

**Figure 1:**
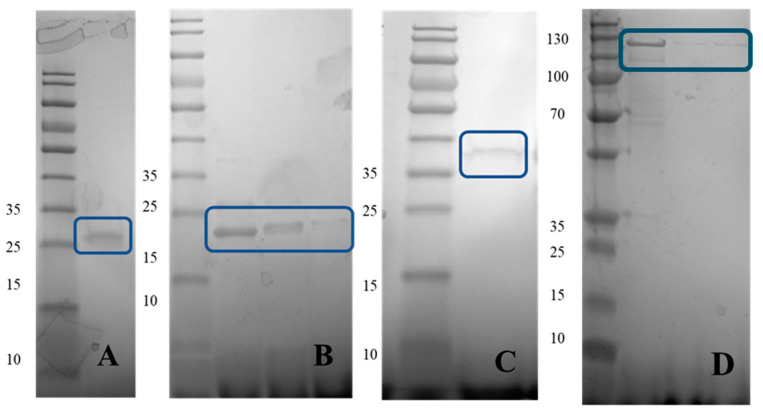
Confirmation of recombinant DUF3916 (**A**), TM2 (**B**), Dam (**C**), and RhsA (**D**) proteins purity using SDS-PAGE. Molecular weight (kDa) of bands in the Page-Ruler Pre-stained Protein Ladder (Thermo Scientific, MAN0011772) are shown to the left of each image.

Recombinant RhsA, Dam, TM2, and DUF3916 proteins were estimated to have a molecular weight of 107.73, 33.62, 20.91, and 21.15 kDa, respectively. Each purified recombinant protein yielded a single band on sodium dodecyl sulfate polyacrylamide gel electrophoresis (SDS-PAGE) that is higher than the expected molecular weight by about 3 KDa for each protein (Figure 1). This is likely due to post-translational modification of the proteins or the addition of amino acids from the expression vector. The identities of the four proteins were confirmed using liquid chromatography ESI MS/MS analysis. The raw mass spectra data were matched to proteins using SEQUEST algorithm of Proteome Discoverer program with the translated ORF database of NRRL B-33077.

### 3.3. Rabbit Serum Antibody Response

**Figure 2:**
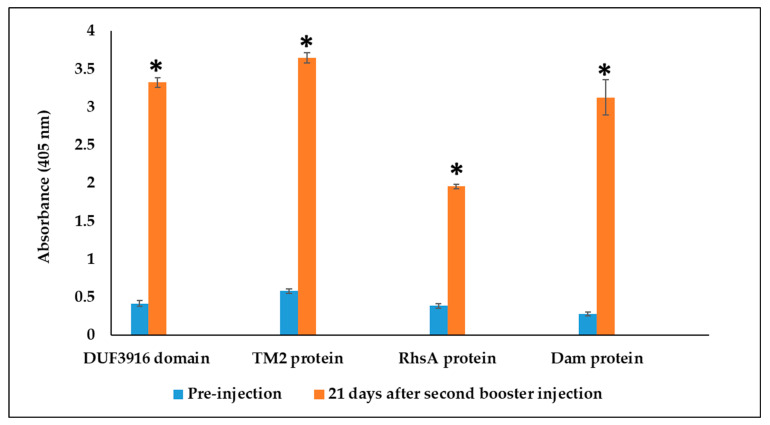
Serum antibody response against recombinant DUF3916, TM2, Dam, and RhsA proteins measured by ELISA using plates coated with respective recombinant protein. Optical densities at 405 nm are means of four replicates of tested serum against the purified protein. Serum was collected 21 days after the second booster injection. Asterisks indicate significantly higher antibody titer compared to pre-injection serum (*p* < 0.0001).

ELISA was used to determine the titers of the polyclonal antibody obtained from each rabbit serum at 21 days after the second booster injection with each of the recombinant proteins. When ELISA plates were coated with purified proteins, sera collected 21 days post-injection showed a significantly (*p* < 0.0001) higher antibody titer than the pre-injected rabbit serum for all four proteins (Figure 2). No significant difference was detected between the two rabbits in the same treatment group in the antibody titer level.

**Figure 3:**
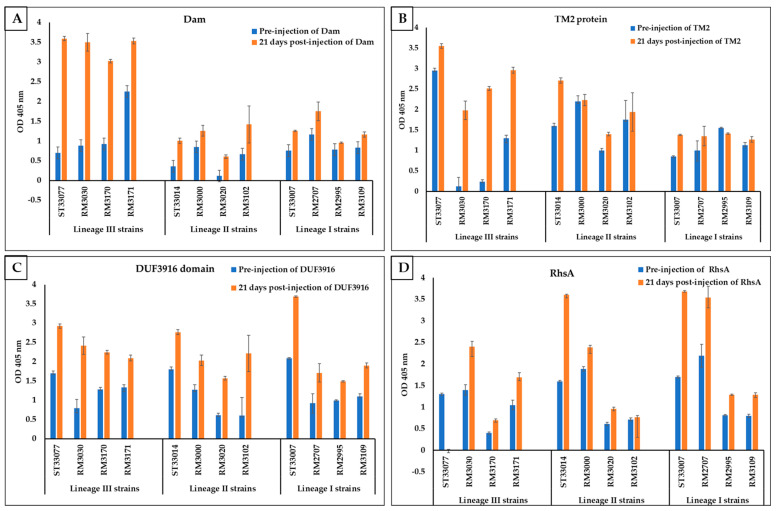
Reactivity of Dam-specific (**A**), TM2-specific (**B**), DUF3916-specific (**C**), and RhsA-specific (**D**) rabbit immune serum against representative strains in lineages III, I, and II. Antibody response was determined by ELISA using plates coated with the killed bacterial suspension of each strain shown using rabbit sera collected 21 days post-injection with purified protein. Optical densities at 405 nm are means of two rabbits with four technical replicates each.

To determine the specificity of polyclonal antibodies for each recombinant protein, the reactivity of immune serum for each protein was determined against the killed bacterial suspension from the twelve *L. monocytogenes* strains (four lineage III strains, four lineage I strains, and four lineage II strains). Pre-injected rabbit sera and sera collected 21 days post-injection with the four purified proteins were tested against the killed bacterial suspension of each representative strain using ELISA (Figure 3). No significant difference was detected between the two rabbits in the same treatment group in the antibody titer level.

To statistically compare the reactivity of immune serum for each recombinant protein to lineage III, lineage I, and lineage II strains, the difference in reactivity between pre-injection serum and immune sera for each *L. monocytogenes* strain was calculated, and the mean difference in reactivity was determined for each protein against lineage III, lineage I, and lineage II. When the mean difference in antibody reactivity for all four lineage III strains was compared to the mean for all four lineage I and all four lineage II strains, significantly higher reactivity was detected in serum against Dam and TM2 proteins in the plates coated with the killed bacterial suspension of *L. monocytogenes* genetic lineage III strains compared with the plates coated with the killed bacterial suspension of *L. monocytogenes* genetic lineages II and I (*p* < 0.0001 and <0.0018, respectively). By contrast, the mean difference in reactivity of serum against DUF3916 and RhsA proteins showed no significant difference between the plates coated with the killed bacterial suspension of *L. monocytogenes* genetic lineage III strains against plates coated with the killed bacterial suspension of *L. monocytogenes* lineage I and II strains (*p* = 0.6 and 0.68, respectively).

When the mean difference in antibody reactivity for each individual lineage III strain was compared to the mean reactivity for all four lineage I and all four lineage II strains using multiple variant comparison, all four lineage III strains (NRRL B-33077, RM3030, RM3170, and RM3171) had significantly higher differences in antibody reactivity against Dam protein compared to the mean difference of all four lineage I strains and all four lineage II strains (Figure 3A). However, TM2 protein only had significantly higher differences in antibody reactivity in RM3030 and RM3170 compared to the mean of all four lineage I strains and all four lineage II strains. As expected, DUF3916 and RhsA proteins did not show any significant difference in antibody reactivity against lineage III strains compared to the mean of antibody reactivity for all four lineage I and II strains (Figure 3B).

### 3.4. Automated Western Blots

**Figure 4:**
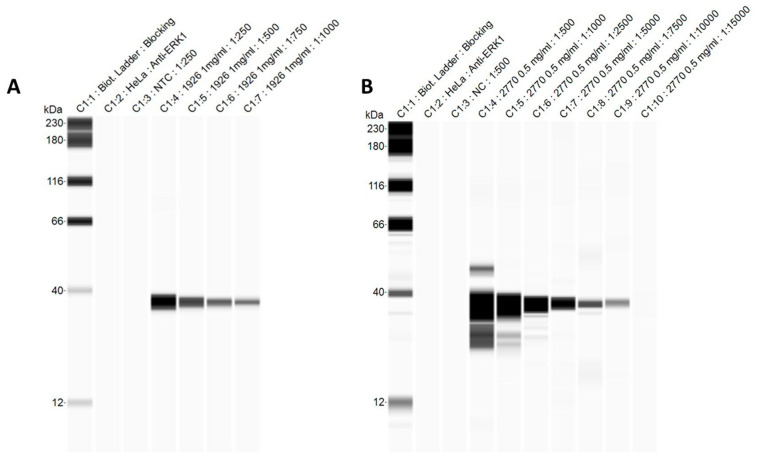
Automated capillary Western blot gel-like image of Dam (**A**) and TM2 proteins (**B**). The predicted molecular weights of Dam and TM2 are 33.62 and 20.9 kDa, respectively. Lane 1: manufacturer molecular weight standard; Lane 2: manufacturer HeLa control; Lane 3: no recombinant protein with immune serum; Lanes 4–7: 5 µg recombinant Dam protein with varying dilutions of immune serum (**A**). Lanes 4–10: 2.5 µg of recombinant TM2 protein with varying dilutions of immune serum (**B**).

Capillary Western blots were conducted to analyze purity and confirm the size of recombinant Dam and TM2 proteins. Compass software generates gel-like images of immunoassays (Figure 4A,B). For Dam, a single clear band was present at 1:1000 and 1:750 serum dilutions, and the size matched the predicted molecular weight for Dam (33.6 kDa). For TM2 protein, a single clear band was visible at 1:7500 and 1:10,000 dilutions of serum. The band was approximately 40 kDa, which is about twice the predicted molecular weight of 20.9 kDa, suggesting the protein may exist as a dimer.

## 4. Discussion

Several subtyping techniques identified four major genetic lineages of *L. monocytogenes* [23,24,25,26], and specific serotypes are associated with each lineage. Lineage I strains are most frequently associated with clinical outbreaks of listeriosis. Lineage II strains also commonly cause clinical listeriosis and are routinely found in food and environmental samples. Lineage III strains are not as common. They are often isolated from animals, and they typically pose little risk of causing disease in humans [23,27].

In addition to serology, differentiation of *L. monocytogenes* lineages currently relies on DNA-based technology. To date, there are no reported biomarkers useful for differentiating *L. monocytogenes* genetic lineages. Since lineage III strains are low risk for human listeriosis, we sought to identify protein biomarker(s) that could distinguish *L. monocytogenes* genetic lineage III isolates from lineage I and II isolates. A protein biomarker could be used for development of a simple antibody-based method for distinguishing lineages. Another objective of the current study was to determine if mass spectrometry-based shotgun proteomics could be used to identify candidate biomarkers. Our reasoning was that mass spectrometry would be an efficient screening method compared to a purely bioinformatics-based search for unique proteins because we could be assured that any candidate biomarker proteins are actually expressed in detectable quantities prior to conducting labor-intensive cloning and expression of candidate recombinant proteins. To test this method, we used three strains of *L. monocytogenes*, one representing each of the three genetic lineages. Electrospray ionization tandem mass spectrometry was used to identify peptides from each of the strains, and comparative proteomics analysis between the three strains yielded candidate proteins unique to lineage III.

Proteome coverage was improved by running liquid chromatography-tandem mass spectrometry in triplicate for each strain. Coverage was further improved by running a liquid chromatography gradient based on molecular weight and running mass spectrometry on the resulting fractions. Increasing the gradient length enhances the number of protein identifications in complex mixtures by reducing the complexity of fractions, which increases the sensitivity of mass spectrometry output [28,29]. Analysis of brain proteome demonstrated that coupling of prefractionation with long gradient LC-MS/MS yielded higher peptide numbers and increased protein coverage with less than 1% protein false discovery [29].

After identification of potential lineage III-specific proteins based on mass spectrometric analysis of three strains, an additional bioinformatic analysis was conducted to help select the best candidate proteins for cloning and expression. BLAST analysis of each candidate lineage III-specific protein against a proteome database of >80 *L. monocytogenes* strains (representing all three lineages) was conducted to account for strain sequence variation within each genetic lineage and select candidate proteins consistently present in lineage III strains but with low percent identity and coverage against all lineage I and II strains. The predicted functions of the four candidate proteins include Dam (NRRL B-33077_1926), RhsA protein (NRRL B-33077_1129), TM2 protein (NRRL B-33077_2770), and hypothetical DUF3916 domain-containing protein (NRRL B-33077_1897).

The protein encoded by TM2 domain-containing protein is predicted to contain two transmembrane domains that are linked by a short loop. There is a conserved DRF (aspartate-arginine-phenylalanine) motif inside this loop, which is seen in several G-protein coupled receptors [30]. There are three TM2 domain-containing genes (TM2D1, TM2D2, and TM2D3) with unknown molecular functions [31]. The TM2D1 protein shares sequence and structural similarities to the beta-amyloid binding protein (BBP) [32]. Several researchers used membrane proteins as biomarkers for differentiating infectious *L. monocytogenes* strains from non-infectious strains and CNS-related strains from non-CNS related strains [24].

DNA methylation at adenine residues by Dam controls the timing and targeting of important biological processes such as DNA replication, chromosome segregation, methyl-directed mismatch repair, and transcription of certain genes [33,34]. Dam DNA methylation modulates the expression of various genes and has a role in pathogenicity of numerous bacterial species, including *Salmonella enterica* [34,35], *Vibrio cholerae* [36], and *Haemophilus influenzae* [37]. Dam proteins are potentially useful as biomarkers in cancer cell detection and disease-associated changes [27,28,29,30,31,32,33,34,35,36,37,38].

Protein RhsA is a member of the *Rhs* family and consists of a complex genetic sequence [39]. The *rhs* genes were first described in *E. coli*, and they were originally thought to be rearrangement hot spots due to a recombination event between nearly identical sequences within the *rhsA* and *rhsB* genes [39,40]. Despite the broad distribution of Rhs family proteins in both bacteria and eukaryotes, the function of this protein is not well studied. Some studies suggest that some Rhs systems encode toxin/immunity protein pairs, and bioinformatic analyses support that Rhs proteins contain toxic nuclease domains [41,42]. In Gram-negative and Gram-positive bacteria, Rhs functions as an intercellular competition mediator by inhibiting the growth of neighboring bacteria [43]. Rhs also functions as an immunity protein to protect bacteria from auto-inhibition [43]. Cloning, expression, and purification of the four-candidate lineage III-specific proteins were very effective using pET28a (5369 bp) in *E. coli* with the T7 promoter. Cloning of the gene encoding RhsA was difficult due to its size (approximately 10 kbp), which is almost twice the plasmid size. Therefore, approximately 2900 bp was cloned in pET28a expression vector, which represents the rearrangement hotspot (RhsA) repeat domain. RhsA is a large protein complex and consists of some distinct sequence features including a GC-rich core (core open reading frame [ORF]), an AT-rich extension (ext-a1) of the core ORF, and an AT-rich region following the core extension (dsORF-a1) [44].

Recombinant RhsA, TM2, and DUF3916 proteins showed a high level of expression in BL21(DE3) *E. coli* in the insoluble fraction. However, Dam protein showed no expression after IPTG induction. Additionally, bacterial growth stopped immediately after IPTG induction. Therefore, we used C41(DE3) *E. coli*, which is more resistant to some toxic proteins, to express Dam, where it was isolated from inclusion bodies.

A lineage III-specific protein biomarker has the potential for development of a simple, field-deployable antibody-based detection method. To begin testing the feasibility of this approach, we developed polyclonal antibodies for each of the recombinant proteins. Some background reactivity was detected against *L. monocytogenes* in all the rabbit sera, which may be due to environmental exposure. *L. monocytogenes* is common in the environment and can colonize rabbit’s intestine. Another factor likely contributing to the background antibody reaction is that the ELISA plates were coated with *L. monocytogenes,* the killed bacterial suspension; therefore, some reactivity could occur from other bacterial antigens.

Significant antibody titers were detected in ELISA using plates coated with the four respective recombinant proteins compared to the pre-inoculation serum. Next, to determine the specificity of the polyclonal antibodies for detection of lineage III *L. monocytogenes*, each was tested for reactivity against whole bacteria from the three original strains (NRRL B-33077, NRRL B-33014, and NRRL B-33007) and nine additional *L. monocytogenes* strains representing the three lineages. Based on ELISA results using four representative strains from each genetic lineage, Dam and TM2 are promising protein biomarkers for development of an antibody-based method to differentiate *L. monocytogenes* genetic lineage III from lineages I and II. Of these, Dam has higher potential than TM2 protein as a biomarker because Dam-specific serum reacted significantly higher against each of the four individual lineage III strains compared to the mean reactivity against all four lineage I and lineage II strains. Western blots based on capillary electrophoresis confirmed that the polyclonal antibodies detected the recombinant Dam and TM2 proteins. The size of Dam was also confirmed (33.62 kDa). Western blot results suggest that TM2 protein (20.9 kDa) exists as a dimer, but little information is published about this protein; therefore, this could not be confirmed. Capillary Western blots provided good sensitivity and protein separation compared to traditional Western blots, and it reduces time and reagent requirements [45].

In conclusion, our results confirmed that Dam and TM2 domain-containing protein are promising candidates as protein biomarkers for differentiating *L. monocytogenes* genetic lineage III from lineages I and II, which will facilitate the identification of *L. monocytogenes* subgroups in diagnostic microbiology laboratories and potentially in food processing facilities. This increased discriminatory capability would assist in assessment of risk from *L. monocytogenes* isolates and accelerate epidemiological investigations. The approach described in the current study shows promise for identifying protein biomarkers for *L. monocytogenes* genetic lineages I and II.

## Figures and Tables

**Table 1:** *Listeria monocytogenes* strains used in this study.

Strain No.	Serotype	Genetic Lineage	Source	Reference
NRRL B-33077	4a	III	Unknown	USDA-ARS
RM3030	4c	III	Bull	[19]
RM3170	4c	III	Unknown	[19]
RM3171	4a	III	Unknown	[19]
NRRL B-33014		II	Unknown	USDA-ARS
RM3000	1/2c	II	Soil	[19]
RM3020	1/2c	II	Unknown	[19]
RM3102	1/2a	II	Monkey	[19]
RM3109	1/2b	I	Unknown	[20]
NRRL B-33007		I	Unknown	USDA-ARS
RM2707	1/2b	I	Cheese	[20]
RM2995	1/2b	I	Cow brain	[19]

**Table 2:** *Escherichia coli* strains and plasmids used in this study.

*E. coli* Strain or Plasmid	Description	Reference or Source
NovaBlue BL21(DE3) C41 (DE3)	*endA1 hsdR17* (rK12–mK12+) *supE*44 thi-1 *recA1 gyrA*96 relA1 *lac* F′[proA+B+lacIqZΔM15:Tn10] (Tet^R^) F−*ompT hsdS gal*; expression host F–*ompT hsdSB (rB-mB-) gal dcm* Derived from Bl21 (DE3) to help plasmid stability	Novagen EMD Millipore Lucigen
pET-28a pET28a_1897 pET28a_2770 pET28a_1129 pET28a_1926	Expression vector; Km^R^ pET28a with cloned ST33077_1897 pET28a with cloned ST33077_2770 pET28a with cloned ST33077_1129 pET28a with cloned ST33077_1926	Novagen This study This study This study This study

**Table 3:** Primers used for PCR amplification.

ORF	Primers	Primer Sequence (5′ to 3′)	Restriction Enzymes	Amplicon Size (bp)
NRRL B 33077_1897	Forward Reverse	AAA**GTCGAC**TTTTCCTATAAACCAAA * AAA**GGATCC**GGTATGGGGAATAAGGA	SalI BamHI	534
NRRL B 33077_2770	Forward Reverse	AAA**GGATCC**ATTTTCATGTTTAATAA AAA**GTCGAC**TTGGGCATCATTCGCTT	BamHI SalI	567
NRRL B 33077_1926	Forward Reverse	AAA**GTCGAC**CACCACCCCTGCCTGAT AAA**GGATCC**GGATCCTTTATTCGTGCAATTAA	SalI BamHI	920
NRRL B 33077_1129	Forward Reverse	AAA**GTCGAC**GGCACCGTCTTCGTGGT AAA**GCTAGC**AAGGACCTGAAGACACA	SalI NheI	2900

* Bold letters at the 5′ end of the primer sequence represent the restriction endonuclease site added to enable directional cloning.

**Table 4:** *Listeria monocytogenes* ST33077 proteins with less than 50% %identity × %coverage in lineage I and II strains.

ORF	Lineage I (%Identity × %Coverage) *	Lineage II (%Identity × %Coverage)
ST33077_0135	NS ^†^	NS
ST33077_0258	WP_003740445.1 (47.77)	WP_012951247.1 (47.32)
ST33077_0314	WP_003725659.1 (10.08)	NS
ST33077_0492	NS	NS
ST33077_0493	WP_023550422.1 (19.28)	NS
ST33077_0937	NS	NS
ST33077_1129	WP_061385917.1 (2.68)	NS
ST33077_1328	WP_003725868.1 (28.53)	WP_014602121.1 (28.15)
ST33077_1569	WP_003728189.1 (16.05)	WP_014601042.1 (10.39)
ST33077_1816	NS	NS
ST33077_1897	NS	NS
ST33077_1926	NS	WP_049962080.1 (26.68)
ST33077_2218	NS	NS
ST33077_2271	WP_003724612.1 (8.91)	WP_003731898.1 (8.91)
ST33077_2323	NS	NS
ST33077_2398	NS	NS
ST33077_2419	NS	NS
ST33077_2617	WP_021496264.1 (8.47)	NS
ST33077_2739	NS	NS
ST33077_2770	WP_003728337.1 (9.27)	NS

* Protein ID of the most similar protein in lineage I or II and %identity × %coverage. ^†^ NS, no significant protein matches found by BLAST.

**Table 5:** Predicted functions of candidate lineage III-specific proteins.

ORF	NCBI ID *	Similarity ^†^ (%)	Function
ST33077_0135	NS ^‡^	0	
ST33077_0258	WP_012581893.1	97	hypothetical protein
ST33077_0314	WP_039381474.1	10.8	guanylate kinase
ST33077_0492	WP_070220410.1	100	hypothetical protein
ST33077_0493	WP_070220412.1	100	hypothetical protein
ST33077_0937	NS	0	
ST33077_1129	WP_014589151.1	94.2029	type IV secretion protein RhsA
ST33077_1328	WP_003739362.1	28.534	oxidoreductase
ST33077_1569	WP_070221254.1	100	hypothetical protein
ST33077_1816	NS	0	
ST33077_1897	WP_012582068.1	95	hypothetical protein
ST33077_1926	WP_077954308.1	100	DNA methyltransferase
ST33077_2218	NS	0	
ST33077_2271	WP_070779485.1	9.54548	peptide ABC transporter substrate-binding protein
ST33077_2323	WP_070219794.1	100	hypothetical protein
ST33077_2398	WP_070219932.1	100	hypothetical protein
ST33077_2419	WP_070219871.1	100	hypothetical protein
ST33077_2617	WP_070284542.1	13.1763	hypothetical protein
ST33077_2739	NS	0	
ST33077_2770	WP_003728958.1	90	membrane protein

* Best BLAST hits in NCBI database. ^†^ Similarity between ST33077 protein and its best BLAST hit. ^‡^ NS, no proteins with significant similarity were identified in the NCBI database.

**Table 6:** Number of peptides identified by LC ESI MS/MS for candidate lineage III-specific proteins and percent coverage of proteins by identified peptides.

Protein	Number of Peptides	% Coverage
ST33077_0135	1	19.697
ST33077_0258	1	8.21918
ST33077_0314	1	9.42029
ST33077_0492	7	6.7623
ST33077_0493	1	4.8913
ST33077_0937	3	15.6522
ST33077_1129	1	0.87069
ST33077_1328	12	8.73016
ST33077_1569	7	19.4915
ST33077_1816	1	9.56522
ST33077_1897	3	21.4689
ST33077_1926	31	24.6528
ST33077_2218	1	3.76712
ST33077_2271	1	6.96203
ST33077_2323	16	7.22892
ST33077_2398	1	7.69231
ST33077_2419	4	20.7547
ST33077_2617	1	12.5
ST33077_2739	1	19.1781
ST33077_2770	100	29.8913

## Data Availability

The data that support the findings of this study are available on request from corresponding author. The data are not publicly available due to privacy restrictions.
